# Emergence Time and Skin Melanin Spot Patterns Do Not Correlate with Growth Performance, Social Competitive Ability or Stress Response in Farmed Rainbow Trout

**DOI:** 10.3389/fnins.2017.00319

**Published:** 2017-06-07

**Authors:** Manuel Gesto, Peter V. Skov, Alfred Jokumsen

**Affiliations:** Section for Aquaculture, North Sea Research Centre, DTU Aqua, Technical University of DenmarkHirtshals, Denmark

**Keywords:** stress-coping style, emergence time, rainbow trout, skin pigmentation, dominance, growth

## Abstract

In wild salmonid fish, specific individual behavioral traits have been correlated with the timing of fry emergence from their gravel spawning nests; Early emerging fish display more aggressive behavior and have a higher probability of becoming socially dominant, compared to fish that emerge at a later stage. Apart from aggression and dominance, other behavioral and metabolic traits, such as boldness, metabolic rate, or growth, have also been linked to emergence time. Altogether, the traits of early- and late-emerging fish resemble those of the proactive and reactive stress-coping style, respectively. As proactive fish are considered more resilient to stress, it may be desirable to select these for aquaculture production. However, it is currently unclear to what extent the link between emergence time and stress-coping styles is maintained in the selective breeding of farmed fish. In the present study, eyed eggs from a commercial supplier were hatched, and larvae fractionated according to their emergence time. Later on, juvenile fish from different emergence fractions were subjected to a stress challenge and also tested to evaluate their competitive ability for food. Beyond some slight dissimilarities in the acute stress responses, emergence fraction displayed no correlation with growth rates, or the ability to compete for feed. Within the whole group of fish utilized in the experiments, no relationship between skin melanin spot pattern and growth performance, stress response intensity, or competitive ability was found. Altogether, the differences in physiological traits related to emergence time were not as strong as those found in earlier studies. It is hypothesized, that the origin and degree of domestication of the fish might be partly responsible for this. The predictive value of skin spots or emergence time to infer the fish stress coping style in farmed fish is also discussed.

## Introduction

Recent research has demonstrated that fish individuals, as other vertebrates, differ in the way they cope with stressors (Castanheira et al., 2017). An individual, upon facing a stressor, displays a number of consistent behavioral and physiological responses that are characteristic of that individual. These responses are commonly referred to as the stress-coping style (SCS) (Koolhaas et al., 1999; Larsen et al., 2015). Within a continuous range of SCSs, two well differentiated extremes have been characterized, the reactive and proactive SCS (Larsen et al., 2015). Different behavioral and physiological traits generally appear associated to those different SCSs. For example, reactive fish usually display a more intense activation of the hypothalamus-pituitary-interrenal (HPI) axis upon stress exposure and lower routine metabolic rates than proactive fish, which in turn are usually more active, more aggressive, more prone to taking risks, and, in the case of salmonids, have a higher tendency for social dominance (Castanheira et al., 2017). The characterization of SCS in different species has gained relevance and interest in recent years, due to potential inherent economical and ethical advantages related to fish farming. Since fish with proactive SCS are in general considered more stress resilient, they could potentially be more suitable for use in aquaculture, assuming that better welfare, growth and disease resistance could be achieved. Nevertheless, the applied potential of SCS-screening as a tool for selection or segregation in aquaculture is still under study.

The central mechanisms underlying the different physiological and behavioral traits of fish with different SCS are not well known. Selecting fish according to their differential responses to stress reveals concomitant differences in certain elements of the CNS, known to participate in the control of the HPI axis, such as the CRF system, the mineralocorticoid (MRs) and glucocorticoid (GRs) receptors, and the brain monoamine neurotransmitters dopamine, norepinephrine or serotonin (5-HT) (Backström et al., 2011; Johansen et al., 2011; Winberg et al., 2016). This suggests that individual differences in the CNS pathways controlling the HPI axis, to some extent are linked to the stress coping style. Among the brain monoaminergic systems, the serotonergic system is activated in specific brain regions upon stress exposure in vertebrates, and has been suggested to play a key role in the organization of the stress response (Dinan, 1996; Winberg et al., 1997; Gesto et al., 2013). Genetic variation in the serotonergic system is associated with differences in personality and temperament (Winberg et al., 2016) and is believed to have a key role in the determination of stress coping style in vertebrates (Puglisi-Allegra and Andolina, 2015).

Although it seems clear, that some behavioral and physiological traits of the fish stress response have heritable components (Pottinger and Carrick, 1999; Millot et al., 2014; Ferrari et al., 2016), the relative importance of other factors (social context and other life-history events) as determinants of a particular SCS is not well known (Pottinger, 2006). In wild salmonids, larval emergence time (which can be considered also as the time for first feeding) has been correlated with the SCS. The term emergence time refers to the time at which individual larva display typical swim-up behavior, abandoning the gravel spawning nest. The timing of this event may differ by as much as several weeks for a given spawning nest (Garcia de Leaniz et al., 1993; Brännäs, 1995) and individuals emerging earlier have been shown to display characteristics associated with a proactive SCS, such as being bolder, more aggressive, and having higher metabolic rates than late-emerging individuals (Metcalfe and Thorpe, 1992; Andersson et al., 2013), which usually show traits considered representative of the reactive SCS. However, it is currently unclear, to what extent the coupling between emergence time and SCS is maintained in farmed/domesticated salmonid populations: To date, no consistent evidence for such a relationship has been demonstrated (Thomson et al., 2011; Vaz-Serrano et al., 2011; Andersson et al., 2013). For example, Vaz-Serrano et al. (2011) found no differences in farmed Atlantic salmon in terms of social status, post-stress cortisol levels and basal metabolism between early- and late-emerging individuals.

In addition to physiological and behavioral traits, skin pigmentation, such as melanin coloration, is heritable and has also been associated with SCS in salmonids (Kittilsen et al., 2009; Khan et al., 2016). Atlantic salmon individuals with higher densities of melanin skin spots had a weaker physiological and behavioral response to stress than less-spotted individuals (Kittilsen et al., 2009). Similarly, rainbow trout selected for lower stress-induced cortisol responsiveness also showed a higher density of eumelanin spots (Kittilsen et al., 2009). These findings suggest that skin spot density is correlated with SCS but the actual predictive value of skin spot number in relation to fish SCS is unknown.

The objective of the present study was to test the hypothesis that emergence time and skin pigmentation pattern are reliable predictors of the SCS, and subsequently the overall performance of juvenile rainbow trout reared in captivity. To test this hypothesis, fish larvae were fractionated according to their emergence time (early fraction: first 20% of fish to emerge; intermediate fraction: mid 20%; late fraction: last 20%). Later, juveniles from the different emergence fractions were co-reared and tested for growth performance under different feeding regimes. Furthermore, their competitive ability for food and their physiological response to an acute stress challenge were also evaluated, taking into account both emergence time and skin melanin spot patterning when analyzing the data to detect any correlation among emergence time, pigmentation pattern, acute stress responsiveness, growth and competitive ability.

## Materials and methods

### Fish and housing conditions

Rainbow trout (*Oncorhynchus mykiss*) eyed eggs (15,000) were purchased from a local hatchery (Piledal Dambrug, Vejle, Denmark) and transferred to the facilities of the Technical University of Denmark (DTU) at North Sea Centre in Hirtshals, Denmark. The eggs came from a rainbow trout population whose selective breeding program has been maintained for over 20 generations. The eggs were kept in incubation trays at 10°C in a current of oxygen saturated water. After hatching, larvae were kept in those trays until the first individuals started to develop active swimming. At that point the larvae were moved to artificial gravel nests, in which they remained sheltered by golf balls simulating natural gravel. Those artificial nests worked as a screening device to separate fish according their emergence time: when the fish started to emerge and swim upwards looking for food, a surface current would sweep them to a separate container (see Vaz-Serrano et al., 2011 for a description of the screening device). For the duration of the swim up behavior, larvae were sequentially removed from the container, counted, classified according to emergence time and moved to a new facility. Three different groups were retained according to their emergence time: an early fraction consisting of the 20% of fish that emerged first; an intermediate fraction, consisting of the 20% of the fish with intermediate emergence time; and a late fraction consisting of the 20% of the fish that emerged last. The remaining 40% of the larvae were not used in the experiments. The total duration of the emergence period was 9 days at 10°C, in accordance to what has previously been reported (Andersson et al., 2013). Each emergence fraction was reared in separate tanks at 12°C for several months until the beginning of the experiments described below. The use of fish in this study complied with Danish and EU legislation (Directive 2010/63/EU) on animal experimentation and was approved by the Animal Welfare committee of DTU Aqua.

### Experiment 1: fish performance under different feeding regimes

In order to compare the growth and competition performance of fish from different fractions, a number of individuals from each of the fractions were selected, grouped together and exposed to different feeding conditions. This experiment was done in April 2016, when the fish were 10 month old and the average mass was 63.7 ± 7.1 g (mean ± SD). At this stage, the different emergence fractions were not different in terms of fish mass, length or condition factor (data not shown). Fifteen individuals from each fraction were PIT tagged under anesthesia (benzocaine solution, 50 mg L^−1^) and co-habited in a 600 L polyethylene tank in a moderate circular current. Experiments were performed in triplicate, using a total of 135 fish (45 per emergence fraction). Fish size and mass were recorded and only fish between 80 and 120% of the general average mass were tagged and used for this experiment. The water (recirculating) was kept at 15°C and its quality (${\text{NO}}_{3}^{-}$, ${\text{NO}}_{2}^{-}$, NH_3_/${\text{NH}}_{4}^{+}$, pH) was controlled daily. Photoperiod was kept the same the fish had had during the previous months (14L: 10D, lights on at 7:00).

After a fasting period of 48 h to recover from tagging/anesthesia, a 15-day growth trial started. Fish were fed for 15 days an increasing amount of commercial feed (EFICO E 920, Biomar, Brande, Denmark) to achieve a daily growth rate of 0.84 throughout the trial, according to the growth model of Rasmussen and From (1991). The food was delivered by automatic belt feeders from 9.00 to 19.00 h. After the 15 day period, the mass and length of each individual was measured.

Specific growth rate (SGR) for each individual was calculated as follows:

SGR = 100^*^(LN(final mass) − LN(initial mass))/number of feeding days.

Immediately after the growth trial, a fasting trial was initiated. The fish were fasted for 10 days and then the mass and length of each individual was evaluated again. The SGR during the 10 day period was calculated for each individual. Finally, when the fasting was over, the fish were re-fed for 7 days at a restricted rate of 0.5% of the fish mass per day, to encourage competition for food. After the 7 day period the mass and length of each individual was recorded and SGR was calculated.

### Experiment 2: competitive ability at low stocking density

During experiment 1 it was observed that, under restrictive feeding, some fish were more successful than others in grabbing food. However, the success of each individual was not necessarily related to its hierarchical level or aggressiveness, since no clear dominance was established in the tanks in terms of space or position in the water column (author's personal observation), probably due to an excess of individuals (45 per tank). Therefore, another experiment was performed, in which the fish were kept at densities favoring the establishment of clear hierarchies, to allow some individuals to actually dominate the feeding of the group. Therefore, 5 fish from each emergence time fraction (differing less than 15% in body mass) were housed together in 600 L tanks for a total of 15 fish per tank. Six replicates of this setup were used. The fish mass was 101.0 ± 8.4 g and the resulting stocking density was approx. 2.5 kg m^−3^. Conditions were kept the same than in experiment 1 with the following exceptions: (i) The number of individuals was 15 fish per tank; (ii) The circular current in the tank was eliminated to avoid schooling behavior.

The trial was started 24 h after allocating the fish. Fish were fed at 0.5% fish mass/day for 7 days. After the 7 day period, the mass and size of each individual was recorded. In this experiment fish were not tagged. The head of each individual was photographed during sizing at the beginning and at the end of the trial and individual identification was done based on the pattern of melanin spots on the head (Merz et al., 2012).

During the experiment, 1–3 fish in each tank could be observed dominating the area surrounding the spot closer to the belt feeder, usually displacing the rest of the fish to the periphery of the tank and/or to depth. Nevertheless, data on dominance interaction was not collected and the growth rate of the animals was used as an indicator of competitive ability in conditions of very low stocking density.

### Experiment 3: acute stress challenge

In order to evaluate any emergence time-related difference in the fish response to stress, fish from the different fractions were grouped together (10 fish from each fraction per tank, a total of 30 fish per tank) in six 600 L tanks. Photographs of the head and from the latero-dorsal area of the fish were taken when sizing the fish. The fish mass was 170.6 ± 28.6 g. Fish were left to acclimate to the tanks for 1 week in the same housing conditions described before and were fed at 1.5% fish mass/day. For the acute stress challenge two tanks were used per day during 3 consecutive days, as follows. Four fish (controls) were quickly netted from one tank, anesthetized (benzocaine solution, 200 mg L^−1^) and sampled. Immediately after netting the controls, the remaining fish in the tank were chased for 2 min with the same net. One and four hours after the chasing protocol, 4 fish were netted again from the same tank and sampled. During sampling, a picture of the head was taken from each individual for identification. Blood (100 μL) was rapidly collected from caudal vessels with ammonium-heparinized syringes. Then, the fish was decapitated and the telencephalon dissected out from the skull and immediately frozen on dry ice for subsequent analysis of serotonergic activity. The blood samples of each batch of fish were immediately centrifuged and the plasma was stored at −80°C for subsequent analyses of cortisol, glucose and lactate. All fish were decapitated within 3 min after being netted. A total of 24 fish (12 from each tank) were sampled per day. The tanks were then left undisturbed for 5 days and then the same protocol was applied for a second round. The fish were fed normally (1.5% fish mass/day), except on the day of sampling. Since fish from the different emergence fractions were mixed in the tanks, the emergence fraction corresponding to each individual was not known during sampling and this resulted in an uneven distribution in the number of samples per fraction and sampling time (**Table 2** and **Figure 4**).

### Biochemical analyses

Plasma cortisol concentrations were measured using a commercial ELISA kit (Neogen Europe, Ayrshire, Scotland, UK), following the manufacturer's instructions. Plasma glucose and lactate were analyzed with colorimetric kits from Sigma (St. Louis, MO, USA).

The levels of serotonin (5-HT) and its main oxidative metabolite (5-hydroxyindoleacetic acid, 5-HIAA) in the telencephalon, were analyzed using high performance liquid chromatography with electrochemical detection (HPLC-EC) as previously described (Gesto et al., 2006), with some modifications. Individual tissues were homogenized in 0.5 mL of a 4% perchloric acid solution. The HPLC mobile phase consisted of 73.9 mM NaH_2_PO_4_, 0.1 mM Na_2_EDTA, and 0.58 mM sodium 1-octanesulfonate in deionized water with a 15.3% (v/v) of methanol. The pH was adjusted to 3.0 with orthophosphoric acid. The chromatographic separation was done at 1.1 mL/min with a Supelcosil™ LC-18-DB column (15 cm × 4.6 mm, 5 μm) from Supelco (Bellefonte, PA, USA). The detection system (ESA Coulochem II detector) included a double analytical cell with oxidation potentials set at 0 mV (first electrode) and +340 mV (second electrode). A conditioning cell at + 400 mV was used before the analytical cell to oxidize potential interferences. The rest of the HPLC system consisted of a Shimadzu LC-10AD pump and a Midas autosampler (Spark Holland, The Netherlands). Acquisition and integration of chromatograms were performed using the Clarity™ HPLC software (DataApex Ltd, Czech Republic).

### Quantification of melanin spots

**Figure 1:**
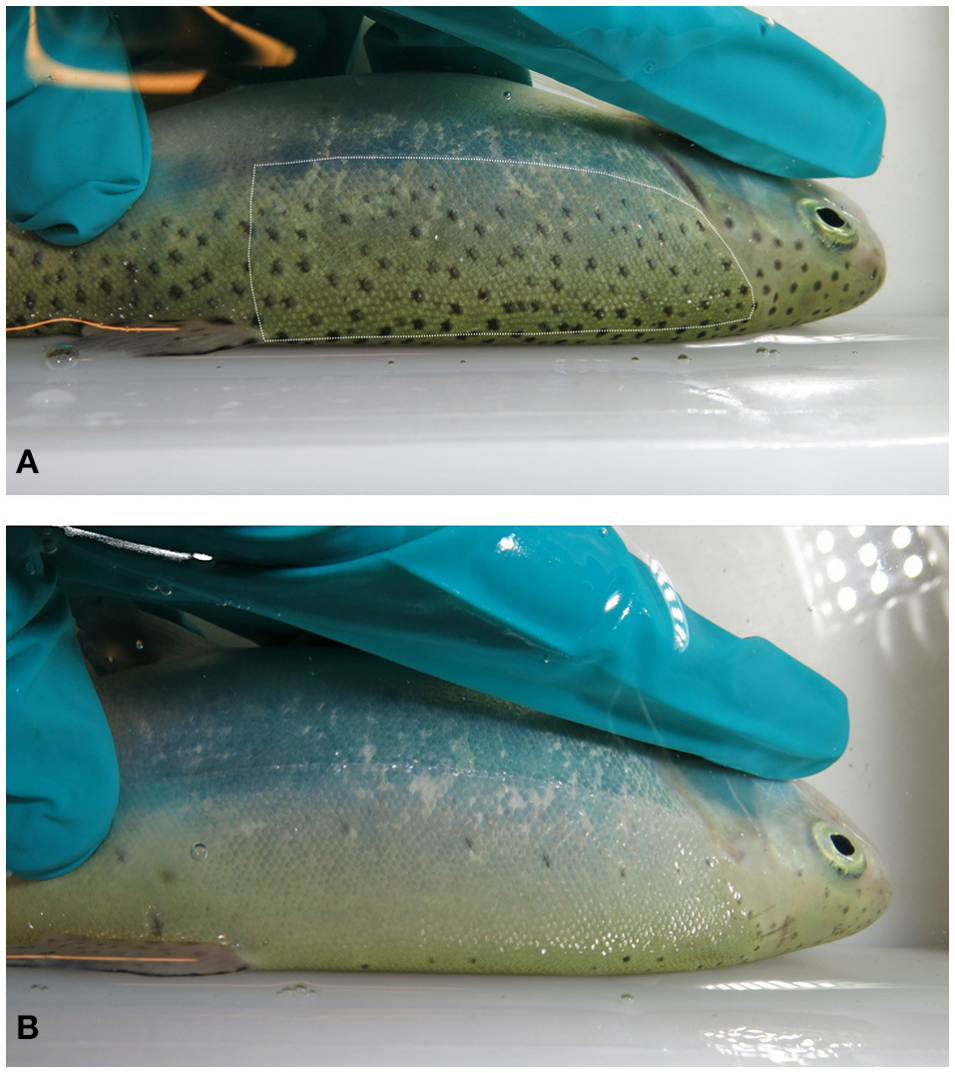
Skin melanin spot pattern in rainbow trout. Pictures show examples for densely spotted- **(A)** and sparsely spotted **(B)** individuals. The dotted line in picture 1 indicates the area used for spot counting, comprising an almost rectangular region between the operculum and the dorsal fin and between the lateral line and the superior anteroposterior midline.

In the performed experiments, a photographic record was kept of each individual in order to identify the individuals and/or to assess the number of melanin spots in the skin. The left latero-dorsal area of the fish, between the operculum and the beginning of the dorsal fin, and between the lateral line and the dorsal anteroposterior midline, was chosen to be quantified for skin spots (Figure 1). Preliminary data showed that this area was a good representative of the number of skin spots in the fish, and that the left and right dorsal parts of the fish were not different in terms of number or size of spots.

### Statistical analysis

SigmaPlot version 12.5 (Systat Software Inc., San Jose, CA, USA) was used for all statistical analyses. The SGRs of the fish of different emergence time fractions under different feeding conditions, as well as the number of melanin spots of fish of the different fractions were analyzed by one-way ANOVA. The relative contribution of each emergence fraction to the 20 best and 20 worst competitors in experiment 2 was analyzed by a Chi-square test. The statistical comparison for plasma and brain stress markers in the acute stress challenge was performed with two-way ANOVA, with emergence fraction and exposure time as factors. Pearson's product-moment correlation was used to assess correlations between skin spot pattern and the fish growth performance. Finally, to assess for any relationship between skin spot pattern and the fish stress response in experiment 3, multiple linear regression analysis was used, with the stress markers as predictor variables and the number of skin spots as the response variable.

## Results

### Experiment 1 fish performance under different feeding regimes

There were no statistical differences in any of the parameters tested among the different replicate tanks.

Fish from the different emergence fractions, showed no differences in SGR after 15 days at a 0.84 daily growth rate (Table 1). Also, there were no differences in terms of growth rate after 10 days of fasting and all groups suffered a similar decrease in mass, as reflected by their SGR. Furthermore, no differences were found among emergence fraction in SGR after fish had been re-fed for 7 days.

**Table 1:** Specific growth rate (SGR) of rainbow trout from different emergence time groups after been co-reared at different feeding conditions.

	**Early**	**Intermediate**	**Late**
Stage 1: 15-day routine growth (daily growth rate of 0.84)	2.60 ± 0.08	2.44 ± 0.09	2.63 ± 0.08
Stage 2: 10-day fasting	−0.73 ± 0.02	−0.73 ± 0.03	−0.76 ± 0.03
Stage 3: 7-day refeeding (0.5% fish mass/day)	0.64 ± 0.10	0.47 ± 0.09	0.62 ± 0.10

*Values are mean and SE of n = 45 fish*.

**Figure 2:**
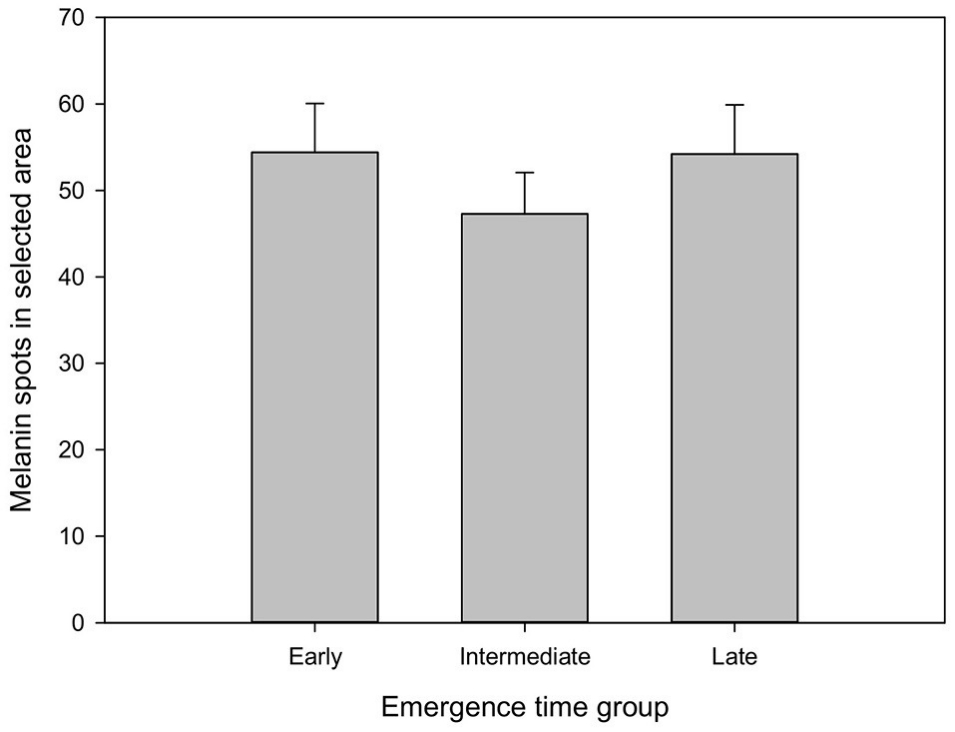
Number of melanin spots of fish from different emergence time groups. Spots were counted in the left dorsal part of each individual (see text and Figure 1 for more detail). Values are mean and SE, *n* = 45 for each column.

Finally, there was no emergence time-related effect in the number of melanin spots (Figure 2), and the number of skin spots showed no correlation with the SGR in any of the stages of this experiment (Table 2).

**Table 2:** Pearson's correlation coefficients for the relationship between skin melanin spots and the specific growth rate (SGR) during experiments 1 (feeding trial) and 2 (competitive challenge), and multiple linear regression *P*-values for the predictive value of plasma and brain stress markers evaluated in experiment 3 (acute stress challenge) for the number of melanin skin spots.

	***n***	**Pearson's correlation coefficient**	***P***	
**EXPERIMENT 1: FEEDING TRIAL**				
Stage 1: 15-day routine growth (daily growth rate of 0.84)				
SGR vs. melanin spots	120	0.145	0.115	
Stage 2: 10-day fasting				
SGR vs. melanin spots	120	−0.105	0.255	
Stage 3: 7-day refeeding (0.5% fish mass/day)				
SGR vs. melanin spots	120	0.111	0.126	
**EXPERIMENT 2: COMPETITIVE CHALLENGE**				
SGR vs. melanin spots	87	−0.0912	0.401	
Multiple linear regression *P*-values				
**EXPERIMENT 3: STRESS CHALLENGE**				
Response variable: number of skin spots				
Predictor variable		Control fish	1 h post-stress	4 h post-stress
Plasma cortisol		0.702	0.256	0.695
Plasma glucose		0.130	0.441	0.592
Plasma lactate		0.850	0.055	0.099
Brain % 5-HIAA/5-HT		0.112	0.927	0.087

### Experiment 2 fish dominance related to competitive ability

**Figure 3:**
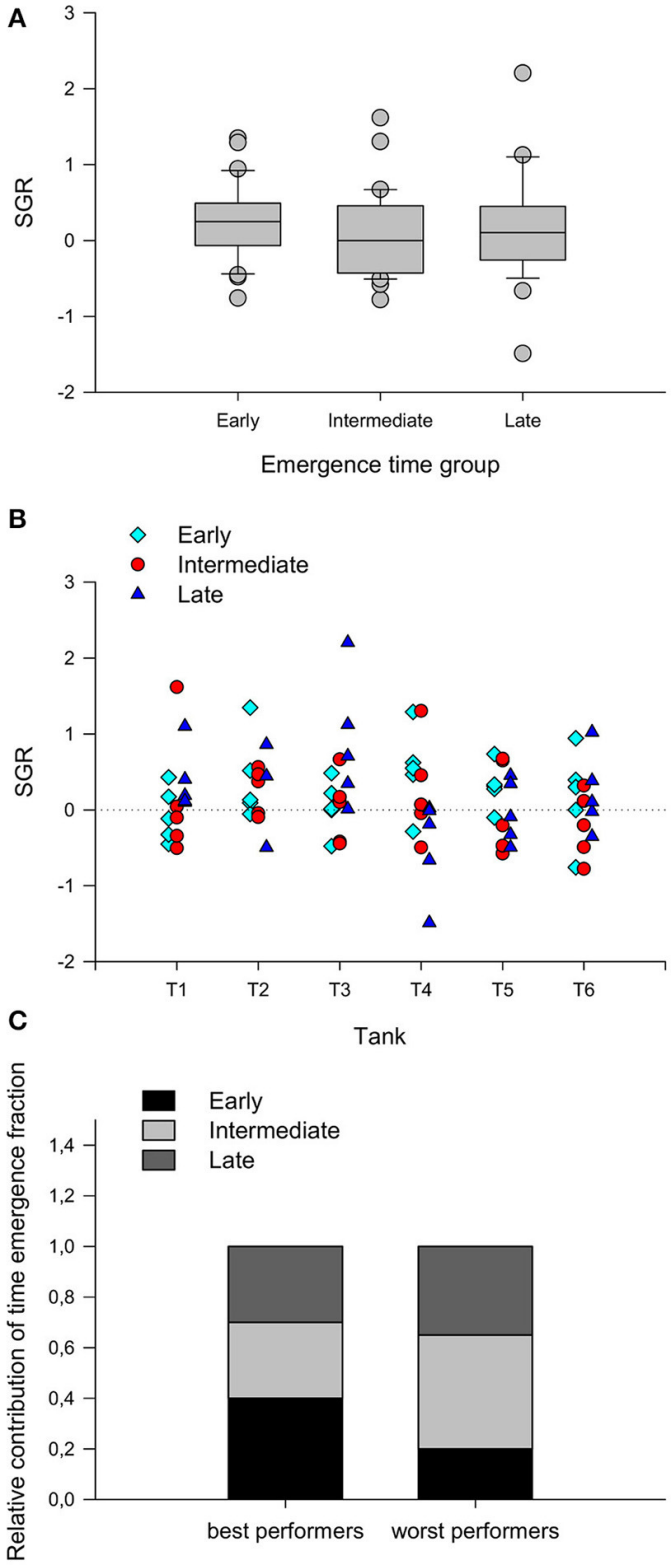
**(A)** Specific growth rate (SGR) of rainbow trout juveniles from different emergence time groups exposed to a 7-day competitive challenge. Fish of the different groups were co-reared at very low stocking density (5 fish of each emergence time group per tank, 2.5 kg m^−3^) to facilitate the formation of social hierarchies (see text for more detail) and were kept for 7 days at a restrictive feed ration of 0.5% fish mass/day. Boxes comprise data from the 25th to the 75th percentiles and error bars indicate the 10th and the 90th percentiles. Lines inside the boxes indicate the median SGR (*n* = 30) and the gray spots represent outliers outside the 10th–90th percentile range. **(B)** Individual SGR values for each fish in the 6 experimental tanks. **(C)** Relative contribution of each emergence time group to the 20 best and the 20 worst performers in the trial.

There were no differences related to the emergence fraction in the ability of each individual to compete for food (Figure 3A). Under the provided conditions of low density and restrictive feeding, between 1 and 3 individuals in each tank dominated food intake and achieved higher SGR (Figure 3B), but no emergence time fraction *per se* showed a higher probability of becoming a better competitor in this situation, when the proportion of fish from each fraction was analyzed within the 20 best and 20 worst performers (Chi-square test, χ^2^ = 2.010; *d.f*. = 2; *P* = 0.366) (Figure 3C).

Furthermore, the count of skin melanin spots in each individual showed no correlation with the SGR during the competitive trial (Table 2).

### Experiment 3 acute stress challenge

**Figure 4:**
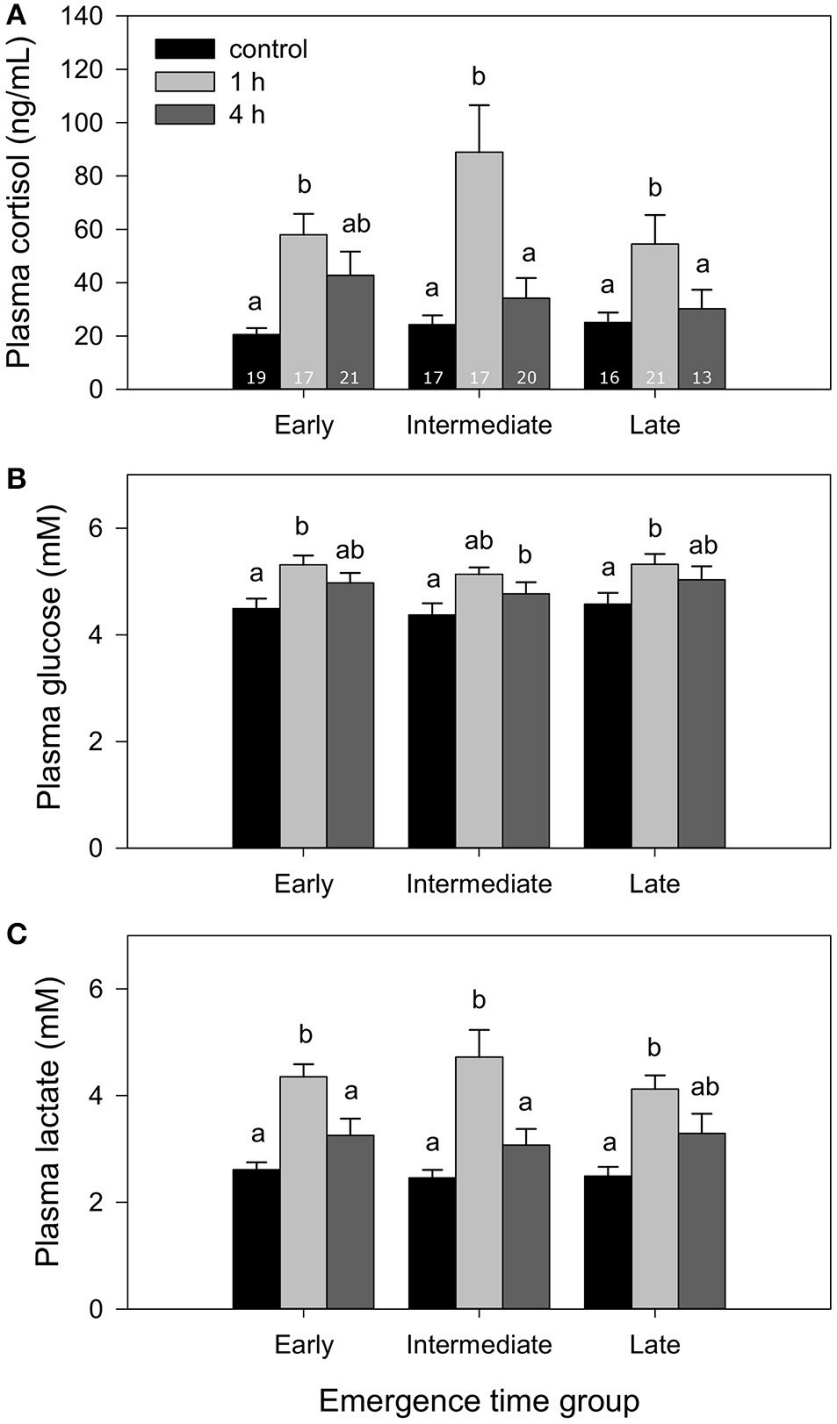
Plasma levels of **(A)** cortisol, **(B)** glucose, and **(C)** lactate in fish from different emergence time fractions (co-reared), at 0 (control group), 1, and 4 h after exposure to a 2-min chasing stress challenge. Bars represent mean and SE (*n* = 13–21, as indicated in **A**). Different superscripts indicate significant differences among time points. No differences were found among emergence time fractions at any time point in any of the plasma stress markers.

Fish from all groups showed a significant response of all three evaluated plasma stress markers, cortisol, glucose and lactate, 1 h after being exposed to a 2-min chase protocol (Figure 4). In all cases, 4 h after the stressor, all stress markers were at levels similar to control fish. No differences were found at any point among emergence time fractions. Similarly, telencephalic serotonergic activity increased 1 h after stress and had recovered control values 3 h later, although the temporary increase did not reach statistical significance in the case of the intermediate group (Table 3). No differences among emergence fractions were found related to serotonergic activation and recovery after stress exposure. The changes in the serotonergic ratio, used as an indicator of the serotonergic activity (Winberg and Nilsson, 1993), were generally sustained by increases in the telencephalic content of 5HIAA (Table 3), since serotonin levels remained unchanged after the exposure to the stressor.

**Table 3:** Telencephalic content (ng g^−1^ wet tissue) of serotonin (5-HT) and its main metabolite 5-hydroxyindoleacetic acid (5HIAA), and values for the ratio % 5-HIAA/5-HT in rainbow trout from different emergence time fractions after exposure to a 2-min stress challenge.

	**Early**	**Intermediate**	**Late**
**5 HYDROXYINDOLEACETIC ACID (5-HIAA)**			
Control	(20) 190.57 ± 12.87	(17) 177.75 ± 12.44^a^	(16) 169.57 ± 10.69^a^
1 h	(17) 220.17 ± 12.66	(17) 221.17 ± 9.91^b^	(23) 214.82 ± 7.81^b^
4 h	(21) 190.60 ± 12.70	(20) 199.39 ± 13.88^ab^	(14) 181.10 ± 12.25^ab^
**SEROTONIN (5-HT)**			
Control	(20) 637.47 ± 25.99	(17) 606.95 ± 25.00	(16) 636.46 ± 35.21
1 h	(17) 625.61 ± 21.11	(17) 710.80 ± 45.33	(23) 652.92 ± 23.06
4 h	(21) 630.88 ± 32.03	(20) 586.76 ± 24.57	(14) 615.47 ± 39.76
**% 5-HIAA/5-HT**			
Control	(20) 29.84 ± 1.48^a^	(17) 28.66 ± 1.31	(16) 27.07 ± 1.21^a^
1 h	(17) 35.31 ± 1.38^b^	(17) 33.10 ± 1.28	(23) 33.57 ± 1.22^b^
4 h	(21) 30.83 ± 1.58^ab^	(20) 31.34 ± 1.72	(14) 30.39 ± 1.87^ab^

*Values are mean and SE of n = 14–21 fish (indicated within parenthesis in every case). Different superscripts indicate significant differences among time points within a given emergence time fraction (Two-way ANOVA, P ≤ 0.05)*.

The number of skin spots in each individual was unrelated to the levels of cortisol, glucose, lactate and serotonergic activity before or after stress (Table 2).

## Discussion

The possibility of selecting fish according their SCS could present an opportunity for improved aquaculture. Not only economical, but also ethical benefits related to the welfare of the farmed fish could arise from a better knowledge of the significance of individual variation/SCSs in fish-farming procedures and facilities. SCS-screening could be a potent tool for a better selection of fish more robust against stress and disease. In the wild, different SCS could offer different advantages and drawbacks for individual survival, which could be one of the reasons why both extreme reactive and proactive SCS have been kept through evolution (Øverli et al., 2007). Under captivity, certain traits associated with the proactive SCS may be advantageous, while traits that are disadvantageous in the wild (e.g., higher exposure to predation) may be irrelevant. From this point of view, there is evidence to suggest that individuals with a proactive SCS could perform better in aquaculture conditions, at least in terms of growth performance, than those of the reactive SCS (Huntingford and Adams, 2005; Martins et al., 2006, 2011; Castanheira et al., 2017). In addition to being considered more robust against acute stressors, there is some evidence to suggest that the proactive SCS is also more robust against certain diseases (Fevolden et al., 1992, 1993; Kittilsen et al., 2012).

One of the main drawbacks in the research dealing with SCS in aquaculture is the current SCS-screening methodology, which is still generally based on individual behavioral and/or physiological testing (Castanheira et al., 2017). The development of high-throughput methods for SCS screening would facilitate this process, and make it applicable in the farming industry. Some methods are under development (see Castanheira et al., 2017 for a review) and are mostly based on punctual behavioral patterns observed after different challenges such as exposure to hypoxia or to other stimuli that can be perceived as threats by the fish (Laursen et al., 2011; Cerqueira et al., 2016; Ferrari et al., 2016; Castanheira et al., 2017). However, the temporal consistency of most of those patterns remains unclear and in some cases, a relatively high degree of randomness could be affecting the screening method. In the present study we tried to evaluate the potential use of emergence time/first feeding behavior and skin melanin-spot patterns as a tool to predict the SCS of rainbow trout juveniles. Those parameters are not based on punctual behavioral observations, but rather on fixed life-history traits related to the development of each individual and would constitute an easy characteristic to develop a high-throughput SCS screening method.

Both tested characteristics have been previously associated to specific SCS in rainbow trout, as well as other species. Previous studies on wild salmonid fish have shown that emergence time/time of first feeding of an individual could be coupled to their SCS (Chandler and Bjornn, 1988; Metcalfe et al., 1995; Larsen et al., 2015). However, whether that relationship is conserved in farmed/domesticated fish is as yet unclear. In rainbow trout lines selected for high or low corticosteroid reactivity against stress, kept in captivity for generations, a tendency was found for the low-responsive offspring to emerge earlier than the high-responsive offspring, suggesting that the link between emergence time and SCS is conserved under domestication (Andersson et al., 2013). In contrast, studies on Atlantic salmon, have failed to demonstrate such a relationship (Vaz-Serrano et al., 2011). The results from the present study show that individual emergence time had no influence on fish performance as juveniles. The three selected emergence fractions performed equally under different feeding regimes: high ration/fast growing, fasting or re-feeding after fasting, suggesting no advantage of selecting one or the other for on-growing in aquaculture. Furthermore, proactive fish usually have higher routine metabolic rates than reactive fish and therefore would be expected to lose more biomass during fasting, but no such differences were found between the early, intermediate and late fractions, suggesting that fish from the different emergence time fractions did not represent different SCSs.

In our second experiment, the ability of each individual to acquire food under conditions of restrictive feeding and very low density was tested and used as a proxy of the hierarchical position of the fish. For salmonids, low stocking density is known to increase the occurrence of aggressive interactions and the formation of dominance hierarchies (Laursen et al., 2015). Under conditions of very low density (15 fish in a 600 L tank), coupled with a restrictive feeding regime, over an extended period of time, the probability that some individuals would dominate the feeding process in the tank, was high. This wouldn't have been possible in conditions of high density or in the presence of a higher number of individuals, where dominant fish could not control the behavior of conspecifics, or in conditions of higher rations, where even the subordinated fish could maintain competition for food, when the more dominant fish became satiated or when many food pellets were administered at the same time. Under our conditions, fish were put in a situation of high social stress, favoring the formation of strong hierarchies and facilitating the dominant behavior of some individuals. After co-rearing fish from the different fractions for 7 days, clear inter-individual differences were seen in SGR. However, no emergence time-related differences in terms of performance or competitive ability were detected. Thus, the lack of an effect of emergence fraction on the probability of the fish to become a better competitor when the conditions for establishment of hierarchies within the tanks were favorable, also suggest that the SCS of the fish was unrelated to their time of emergence.

Lastly, no differences were found among emergence fractions in telencephalic serotonergic activation or in the increase of plasma cortisol, glucose and lactate, following an acute stress challenge. It is known, that the fish stress response is gradable upon exposure to stressors of different duration and/or severity (Gesto et al., 2013), and the present results suggest that the three emergence time groups were equal in the way they perceived the threat and in their subsequent endocrine response to the chasing protocol. In fish, as in other vertebrates, the brain serotonergic system is known to be involved in the regulation of different behavioral aspects related to the animal stress coping style such as aggression and dominance (Larson and Summers, 2001; Lepage et al., 2005; Summers et al., 2005) and also seems to be part of the neuroendocrine cascade that takes place after stress exposure. The serotonergic activity is rapidly stimulated by different stressors in specific regions of the brain (Gesto et al., 2013; Vindas et al., 2016) and this activation has been suggested to participate in stressor recognition and in the subsequent activation of the neuroendocrine stress response (Dinan, 1996; Winberg et al., 1997; Gesto et al., 2013). Differences in serotonergic activity have been found between fish differing in stress responsiveness (Schjolden et al., 2006), and between dominant and subordinate fish (Øverli et al., 1999), suggesting that serotonergic activity may be directly or indirectly linked to stress coping style in fish. Those data, together with evidence from other groups of vertebrates point to a relevant role of the serotonergic system in determining the stress coping style in vertebrates (Puglisi-Allegra and Andolina, 2015). Proactive fish, for example, are considered to be more robust against mild acute stressors, and have been shown to display a lower degree of activation of both brain serotonergic activity and plasma corticosteroids after stress (Øverli et al., 2001; Schjolden et al., 2006). However, a lack of correlation between emergence time and the activation of the HPI axis has been also observed in other studies with salmonids (Vaz-Serrano et al., 2011; Thörnqvist et al., 2015). This lack of consistency between different studies has been observed also in mammals and the consideration of HPI/HPA (hypothalamus-pituitary-adrenal) axis as part of the SCS has been questioned (Koolhaas et al., 2010; Thörnqvist et al., 2015). Nevertheless, the lack of stress responsiveness-related differences in telencephalic serotonergic activity and plasma stress metabolites among fractions in this study also points against our initial hypothesis.

Altogether, the results of this study showed that fish from the different emergence time fractions were not different regarding their SCS, and, that the emergence time/time for first feeding in farmed rainbow trout is not necessarily useful as a predictive tool to infer the SCS or the individual performance. However, it is important to emphasize that the fish used in the present experiments have been selectively bred for generations and probably are highly domesticated, and that the coupling between emergence time and SCS may have lost during domestication (Vaz-Serrano et al., 2011; Andersson et al., 2013). In this respect, different evolutionary mechanisms could explain this decoupling. On one hand, it has been suggested that a performance-based selection of breeders could have caused a selection favoring fish of the proactive SCS (Huntingford, 2004; Sundström et al., 2004). According to this, the current variability in emergence time in fish that have been selected toward the proactive SCS for many generations, could well differ from the variability found in wild populations, and that could be part of the reason why no clear association between emergence time and SCS is found in farmed fish. On the other hand, a lack of evolutionary pressure on time of emergence could have driven a relaxed selection of this trait (Andersson et al., 2013) and resulted in the loss of the SCS-emergence time association.

A phenotypical trait, such as the number of melanin spots in the skin, has also been suggested to be associated with the SCS in salmonid fish (Kittilsen et al., 2009, 2012), in such a way that highly spotted individuals usually show a lower corticosteroid response after acute stress, a trait associated to the proactive SCS. This fact seems to be part of a common relationship that can be found in many vertebrate species, in which extensive melanin-based pigmentation correlates with proactive behavior, and stress- and disease-resistance (Khan et al., 2016). It has been recently hypothesized that in rainbow trout, the link between cortisol levels and melanin-based pigmentation could be mediated by the cortisol-induced stimulation of the agouti signaling protein (ASIP) in the skin, which would in turn inhibit the production of eumelanin, resulting in less, and smaller melanin spots in individuals with sustained higher cortisol levels (Khan et al., 2016). A negative correlation between post-acute stress cortisol and the number of melanin spots has been shown in Atlantic salmon and rainbow trout (Kittilsen et al., 2009, 2012; Khan et al., 2016). However, in the present study, the relationship was clearly non-existent, as was any correlation between basal or post-stress cortisol levels and the individual skin spot patterns. The reason for this is unknown, but could be related to the kind of stress challenge used in the present study, which was a group-based chasing challenge. In this kind of challenge, the basal cortisol level of each individual before the chasing protocol is more difficult to control than in the individual-based confinement tests used in the other studies. Because of that, the post-chasing cortisol response in these fish may not reflect their average cortisol levels during their lifetime, which could be determining the skin melanin pigmentation pattern, and that could partly explain the observed lack of correlation between the cortisol response to stress and the number of skin spots. Our data in this regard also demonstrated that there was no relationship between emergence time and the number of melanin spots. Nor was a correlation observed, between melanin spots and the ability of the fish to compete in a situation of high social stress, as demonstrated by experiment 2. All gathered data about the number of skin melanin spots strongly suggest that the skin pigmentation pattern has no predictive value to infer the SCS of a given individual in the trout population used in this study, invalidating also this part of our initial hypothesis.

In conclusion, this study demonstrated that the relationship between the larval time of first feeding and the SCS, previously observed in wild salmonids, was absent in the population of farmed rainbow trout used in here. Based on these data, either selecting fish based on their individual emergence time as larvae or in the number of skin melanin spots as juveniles has a poor potential as a selection tool to discriminate fish of different stress-coping styles. However, the situation may not be the same for fish of other origins, since other different strategies for selective breeding could have had different impacts in the relationships between emergence time or skin spot patterns and the SCS of the fish.

## Author contributions

MG, PS, and AJ: conceived and designed the experiments, wrote, revised, and accepted the final version of the manuscript. MG: performed the experiments and analyzed the data.

### Conflict of interest statement

The authors declare that the research was conducted in the absence of any commercial or financial relationships that could be construed as a potential conflict of interest.
